# *SimPhy*: Phylogenomic Simulation of Gene, Locus, and Species Trees

**DOI:** 10.1093/sysbio/syv082

**Published:** 2015-11-01

**Authors:** Diego Mallo, Leonardo De Oliveira Martins, David Posada

**Affiliations:** Department of Biochemistry, Genetics and Immunology, University of Vigo, Vigo 36310, Spain

**Keywords:** Gene conversion, gene duplication and loss, gene family evolution, horizontal gene transfer, incomplete lineage sorting, locus tree, simulation, species tree

## Abstract

We present a fast and flexible software package—SimPhy—for the simulation of multiple gene families evolving under incomplete lineage sorting, gene duplication and loss, horizontal gene transfer—all three potentially leading to species tree/gene tree discordance—and gene conversion. SimPhy implements a hierarchical phylogenetic model in which the evolution of species, locus, and gene trees is governed by global and local parameters (e.g., genome-wide, species-specific, locus-specific), that can be fixed or be sampled from a priori statistical distributions. SimPhy also incorporates comprehensive models of substitution rate variation among lineages (uncorrelated relaxed clocks) and the capability of simulating partitioned nucleotide, codon, and protein multilocus sequence alignments under a plethora of substitution models using the program INDELible. We validate SimPhy's output using theoretical expectations and other programs, and show that it scales extremely well with complex models and/or large trees, being an order of magnitude faster than the most similar program (DLCoal-Sim). In addition, we demonstrate how SimPhy can be useful to understand interactions among different evolutionary processes, conducting a simulation study to characterize the systematic overestimation of the duplication time when using standard reconciliation methods. SimPhy is available at https://github.com/adamallo/SimPhy, where users can find the source code, precompiled executables, a detailed manual and example cases.

Recent advances in sequencing technologies have enabled the expansion of genome-wide phylogenetic studies, unveiling not only extensive phylogenomic incongruence (Jeffroy et al. 2006; Salichos and Rokas 2013) but also reviving consideration of how ancestral polymorphisms sort within populations (Edwards 2009). Indeed, it is well known that gene and species phylogenies can be inconsistent due to evolutionary processes like incomplete lineage sorting (ILS), gene duplication and loss (GDL), and horizontal gene transfer (HGT) (Goodman et al. 1979; Pamilo and Nei 1988; Takahata 1989; Maddison 1997; Page and Charleston 1997). Not surprisingly, the gene tree/species tree “dilemma” has received a lot of attention in recent years, and consequently a plethora of species tree reconstruction methods have been published (Chaudhary et al. 2010; Heled and Drummond 2010; Liu et al. 2010; Boussau et al. 2013; De Oliveira Martins et al. 2014; see Mallo et al. 2014a for a review; Mirarab and Warnow 2015). Although several benchmarks of species tree methods have been carried out (Leache and Rannala 2011; Yang and Warnow 2011; Bayzid and Warnow 2013; Mirarab et al. 2014), they have generally focused on single causes of phylogenetic discordance. Nevertheless, ILS, GDL, and HGT can act simultaneously during genome evolution yielding synergistic evolutionary scenarios (Mallo et al. 2014b) and, therefore, it would be convenient to consider them all together. However, none of the state-of-the-art simulation programs is able to yield scenarios that consider these processes at once. Rather, they usually focus on single evolutionary processes, which partially explain why benchmarking studies have usually been restrictive in this regard. Thus, ILS is usually simulated with tools that implement different extensions of the multispecies coalescent model (MSC) (Rannala and Yang 2003), like Mesquite (Maddison and Maddison 2015), MCcoal (Rannala and Yang 2003), or GUMS (Heled et al. 2013). Moreover, for small species trees, structured coalescent simulators like ms (Hudson 2002), SGWE (Arenas and Posada 2014), or scrm (Staab et al. 2015) could be also used. Apart from standalone programs, there are also phylogenetic libraries like DendroPy (Sukumaran and Holder 2010) that include the simulation of ILS. GDL can be modeled using birth–death processes traversing the species tree like in Arvestad et al. (2003), and HGT is usually simulated as a Poisson-distributed series of transfer events, like in HGT_simul (Galtier 2007). Nowadays, only very few tools are able to simulate phylogenies jointly considering multiple sources of phylogenomic incongruence, like PrIME-GenPhyloData (Sjostrand et al. 2013) and DLCoal_sim (Rasmussen and Kellis 2012). The former combines GDL and HGT, while the later considers GDL and ILS. In addition, there are also genome simulators like ALF-A (Dalquen et al. 2012) or EvolSimulator (Beiko and Charlebois 2007), that can integrate several evolutionary processes at the genomic level but do not consider population-level events, like ILS. Altogether, we are not aware of any tool able to simulate phylogenetic trees considering the joint action of ILS, GDL, and HGT.

To facilitate more realistic simulations, we present here a fast and flexible simulation tool—*SimPhy*—that can simulate the evolution of multiple gene families under ILS, GDL, HGT (via homologous replacement), and gene conversion (GC). *SimPhy* implements a flexible hierarchical parameterization scheme that considers genome-wide and gene family-specific conditions, including different sources for evolutionary rate variation among lineages. Moreover, these parameters can be fixed or sampled from statistical distributions defined by the user. In addition, *SimPhy* does not only generate gene trees, but it is also able to produce multilocus sequence alignments in a subsequent step using INDELible (Fletcher and Yang 2009). These features make *SimPhy* a powerful tool to understand the interaction among ILS, GDL, HGT and GC or for a comprehensive benchmarking of phylogenomic methods.

## Simulation of Gene, Locus, and Species Trees with Simphy

**Figure 1.**
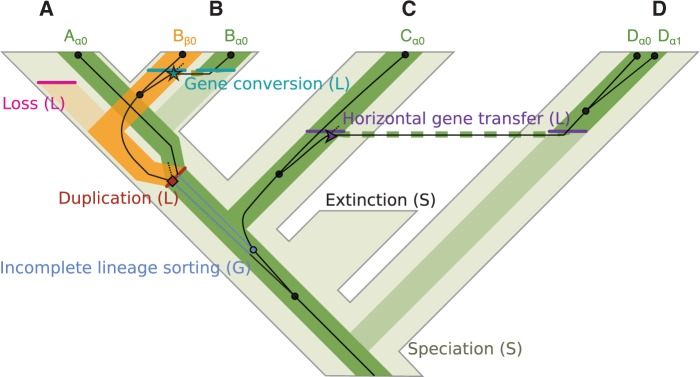
Three-tree model and evolutionary events simulated in *SimPhy*. The figure shows a species tree (thick light tree in the background) that embeds a locus tree (medium-thick lines) that includes a gene tree (thin dark lines with nodes represented by black dots). The sample consists of six gene copies (${\text{A}}_{\alpha 0}$, ${\text{B}}_{\beta 0}$, ${\text{B}}_{\alpha 0}$, ${\text{C}}_{\alpha 0}$, ${\text{D}}_{\alpha 0}$, and ${\text{D}}_{\alpha 1}$) that belong to two loci (α and β) in four different species (A, B, C and D) and five individuals (${\text{A}}_{0}$, ${\text{B}}_{0}$, ${\text{C}}_{0}$, ${\text{D}}_{0}$, and ${\text{D}}_{1}$). Letters in parenthesis indicate at which phylogenetic level an event takes place (S = species tree, L = locus tree, G = gene tree). Coalescent bounds generated by GDL, HGT, or GC events are indicated with an icon (duplication: square, HGT: arrow, GC: star). Dashed lines in the locus tree, generated by HGT and GC, link locus tree branches that are depicted apart for clarity. Lighter locus tree branches represent lost branches due to gene loss or locus replacement by HGT or GC. Dashed gene tree branches indicate lost/unsampled lineages in the original locus from the individual where the duplication took place.

*SimPhy* simulates the evolution of multiple gene families under a hierarchical phylogenomic model in which gene trees evolve inside locus trees, which in turn evolve along a single species tree (Fig. 1). While the three-tree model was first proposed by Rasmussen and Kellis (2012), who implemented it in the program DLCoal_sim, *SimPhy* extends this approach in multiple ways. Apart from ILS and GDL, *SimPhy* also jointly considers HGT and GC, plus species extinction. Furthermore, GDL, HGT, and GC rates are allowed to vary among gene families. *SimPhy* also relaxes the assumption of a strict molecular clock and implements different sources of rate heterogeneity among lineages at the species, gene family and gene tree level. Moreover, the generation time is allowed to vary along the species tree, incorporating an additional layer of rate variation. *SimPhy*'s simulation parameters can be sampled from user-specified distributions at the species, locus, and gene tree level, and in some cases made interdependent. This allows users to carry out simulations following a very flexible strategy in which each parameter is defined by a prior distribution that can represent specific, biologically relevant scenarios. Nevertheless, (classic) simulation studies based on combinations of fixed, discrete parameter values can also be easily implemented.

### The Three-Tree Model

*SimPhy* implements and extends a hierarchical phylogenetic model (Rasmussen and Kellis 2012) that considers three different layers: species, locus, and gene trees.

#### Species trees

Depict the evolutionary history of the sampled organisms. Species tree nodes represent speciation events, while the branches reflect population history: branch length and width represent elapsed time and effective population size (Ne), respectively. Note that we consider species as a diverging interbreeding structure regardless of any taxonomic rank. Therefore, species trees might be equivalent to population trees when the organismal units of interest are conspecific populations.

#### Locus trees

Represent the evolutionary history of the sampled loci for a given gene family. Since the loci exist inside individuals evolving as part of a population, the locus tree is contained within the species tree. In a locus tree the nodes depict either genetic divergence due to speciation in the embedding species tree or locus-level events like duplication, loss, HGT, or GC, while branch lengths and widths represent time and Ne as before.

#### Gene trees

Represent the evolutionary history of the sampled gene copies, which evolve inside a locus tree. Gene tree nodes indicate coalescent events, which looking forward in time correspond to the process of DNA replication and divergence, and which in the absence of migration and HGT can only occur before the speciation time. The lengths of the gene tree branches represent the expected number of substitutions per site.

### Time Units

*SimPhy* simulates species, locus, and gene trees in continuous time. Species and locus trees are measured in number of generations, while gene trees are measured in expected number of substitutions per site. The number of generations can be related to absolute time if a generation time (in number of generations per time unit) is specified, while the number of substitutions per site can be related to the number of generations given a specific substitution rate (expected number of substitutions per generation). Thus, rates of events that take place on the species tree (e.g., speciations) are measured in number of events per absolute time unit, while locus-specific rates (e.g., duplications) are measured in number of events per generation.

### Evolutionary Processes Implemented

*SimPhy* defines and implements different evolutionary processes as follows:

#### Speciation

Separation of an ancestral species into two new species that do not interbreed. Speciation events are represented by nodes in the species tree, and can also be reflected in the locus tree.

#### Extinction

Disappearance of a species. Extinctions take place during the simulation of the species tree, affecting its final topology, but are not considered further.

#### Gene duplication

Copy of a locus into an unlinked location in the same genome. Gene duplication events generate nodes in the locus tree. We do not consider duplication polymorphisms (see below).

#### Gene loss

Deletion of a locus. Losses generate locus tree leaves that do not reach the present, and that are not considered during the gene tree simulation process. We do not consider loss polymorphisms.

#### HGT

Copy of a locus into a different, contemporary species. We assume that the homologous locus in the receiving species is replaced by the transferred locus (i.e., replacing HGT). Each transfer generates two nodes in the locus tree, one representing the loss of the replaced locus (receptor) and another showing the incorporation of the transferred lineage. We do not consider transfer polymorphisms. Transfers from extinct lineages (see e.g., Szollosi et al. 2013), or additive transfers (those generating a new locus in the receiving genome) are also not considered in the current version of the model.

#### GC

Replacement among homologs within a species. Each GC generates two nodes in the locus tree, one representing the loss of the replaced locus (receptor) and another showing the incorporation of the converted lineage. We do not consider GC polymorphisms.

#### Lineage sorting

Consideration of the coalescent history of the sampled gene copies, allowing their history to be incompatible with the species tree history. It is implicitly reflected at the gene tree level, and can be spotted when mapping locus and gene tree nodes.

These evolutionary processes are modeled in *SimPhy* using a mixture of existing and original strategies. The *species tree* is simulated (or user-defined, see section `Simulation Process') by either a Yule (Yule 1925) or a birth–death (Kendall 1948) process, considering speciations and extinctions. At this point, *SimPhy* only considers extant species. Each *locus tree* is described by a constant-rate birth–death process that considers duplication and losses, coupled with another two pure-birth processes that describe the HGT and GC events. Regarding the last two, *SimPhy* incorporates two variants, one in which the receptor is randomly chosen from the candidates and another that takes into account the evolutionary distance between donors and candidate receptors—that is, with reception probability inversely proportional to the phylogenetic distance.

**Figure 2.**
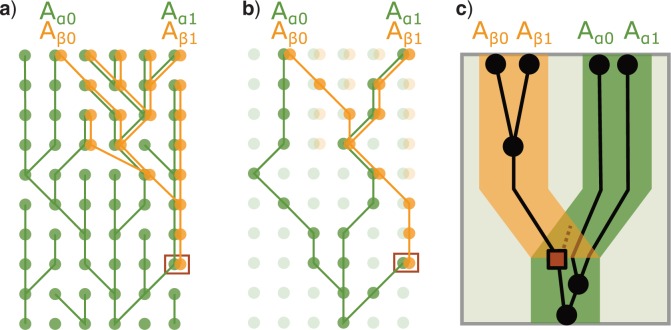
Coalescent bound enforced by duplication. The figure represents the evolutionary relationships of four gene copies (${\text{A}}_{\beta 0}$, ${\text{A}}_{\beta 1}$, ${\text{A}}_{\alpha 0}$ and ${\text{A}}_{\alpha \text{1}}$) belonging to two individuals (${\text{A}}_{0}$ and ${\text{A}}_{1}$) of the same species (A) with two loci (α and β). The locus β was originated by duplication in the individual surrounded by a square. We assume that the duplication occurs initially in one individual, and afterward spreads throughout the whole sample. Thus, lineages coming from the β lineage cannot exist below the duplication event, imposing a coalescent bound (i.e., they have to coalesce before the duplication event [above in this figure]). The three subfigures represent alternative views of the same scenario. Subfigure a) depicts the relationships of the whole population, while in subfigure b) only the sampled lineages are highlighted. Finally, subfigure c) shows the three-tree representation of the sample genealogy.

Duplications, HGTs and GCs act as coalescent bounds for the subtree that corresponds to the new/replaced locus (the “bounded subtree”), since these events initially affect a single individual and only afterward may become fixed in the population (Fig. 2). Nevertheless, *SimPhy* does not consider locus polymorphisms. We assume instead that duplications, HGTs and GCs spread fast enough in the population so all descendant individuals in the sample carry them, or alternatively, that they drift away so quickly that are absent in the sample. The result of this assumption is that every sampled individual from a particular lineage and gene family carries the same number of gene copies. Otherwise, we would have been obligated to include a population genetics model for the evolution of gene copy polymorphisms, which seemed out of scope.

*Gene trees* modeled by the multilocus coalescent model (Rasmussen and Kellis 2012) are expanded to consider bounded subtrees generated by HGT and GC. This model is built upon the MSC (Rannala and Yang 2003) and, therefore, inherits the same assumptions: each branch of the container tree is composed of a perfect Wright–Fisher population (constant effective population size, non-overlapping generations, random mating, neutrality), no recombination within loci and free recombination between loci. Consequently, all of the evolutionary processes except speciation and extinction are considered independently for each gene family. Some of these assumptions might be relaxed in future versions of *SimPhy*, for example, allowing migration of individuals between species and/or recombination within loci.

### Simulation Process

#### Species trees

Sampled either using a pure-birth (Yule) model or a birth–death model, parameterized by birth (speciation) and death (extinction) rates (given in number of events per generation) and either number of leaves, tree height or both. The simple sampling approach algorithm (SSA) is used when the number of leaves is not specified, while the birth–death rate sampling approach (BDSA) algorithm is used otherwise (see Hartmann et al. 2010 for more details). An out-group species can be added to the resulting species tree before the simulation of the locus trees.

#### Locus trees

Simulated using a SSA to sample a birth–death process—describing duplications and losses—coupled with two additional pure birth processes—describing HGTs and GCs—along each species tree branch, in a preorder fashion (from the root to the tips). This is followed by a second preorder traversal that samples receptors from contemporary candidates for each HGT/GC, completing the appropriate SPR branch rearrangement to generate the definitive locus tree.

#### Gene trees

Locus tree branches that do not pertain to bounded subtrees (i.e., representing the existing locus before any bounding duplication/HGT/GC event) are modeled by the multispecies coalescent, and, therefore, sampled by successive coalescent simulations along each branch of the locus tree, traversed in post-order in the opposite direction than the locus tree simulation. For each locus tree branch, a standard coalescent simulation starts with a number of lineages that enter the locus tree branch toward the root, terminating when the end of the locus branch is reached, or when there is only one lineage left. On the other hand, the simulation of gene trees along bounded locus subtrees implies a much more complex strategy, based on sampling the number of gene tree lineages going through every node of the locus tree, and obtaining the coalescent times conditioned on these numbers. This approach requires the calculation of gene tree lineage count probabilities for every branch of the these locus subtrees, for which *SimPhy* uses a dynamic programing algorithm modified from the one proposed by Rasmussen and Kellis (2012). A pre-order traversal (from the root to the tips) is then conducted to sample the number of lineage counts going across every locus subtree branch, using the inverse transform sampling method with the cumulative distribution function (CDF) of the number of input gene tree lineages—those entering the locus tree branch towards the root—conditioned on the precalculated gene tree lineage count input probabilities, output lineage counts (those leaving the locus branch toward the root), effective population size, and branch length for every branch of the considered bounded locus subtree (Online Appendix 1 available as Supplementary Material on Dryad at http://dx.doi.org/10.5061/dryad.707td). This procedure starts at the root of each bounded locus subtree, where the number of output lineages is fixed to one—because of the bound imposed by the duplication/HGT/GC event—and then samples the counts along the bounded locus subtree stopping at its leaves. With the lineage counts already set, the coalescent times are sampled using the inverse transform of the CDF of the coalescent times conditioned on lineage counts as in Rasmussen and Kellis (2012).

### Distribution-Driven Parameterization

**Figure 3.**
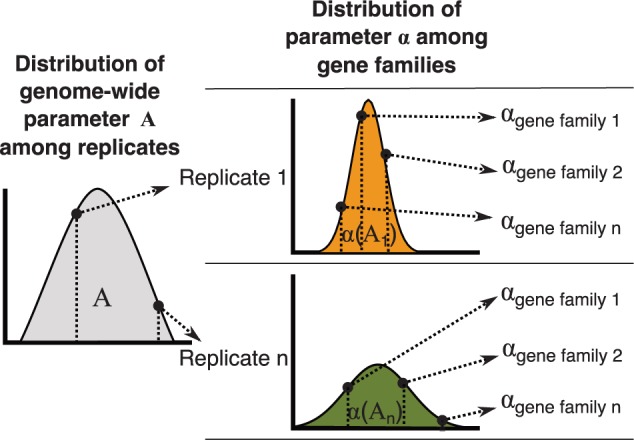
Distribution-driven parameterization. For every simulation replicate parameter A is sampled from a given distribution. The sampled value of A will shape the distribution among gene families of our parameter of interest, α, for that replicate (i.e., A acts a hyperparameter for α). Then, for every gene family the α parameter is sampled from the resulting distribution to determine its evolution. This particular schema can be applied to all gene family-specific parameters like duplication, loss, HGT and GC rates. Similar schemas with more or less layers can be applied to other simulation parameters. Different statistical distributions can be specified by the user (e.g., Uniform, Normal, Exponential, Gamma).

While most simulation studies are parameterized using a grid consisting of combinations of discrete parameter values, *SimPhy* has the capability of sampling parameter values from prior statistical distributions (as in Darriba et al. 2012; see also Leigh and Bryant 2015). Such distributions are defined by the user, and currently include Uniform, Normal, Lognormal, Exponential, and Gamma plus the possibility of fixing parameter values. Different parameters are sampled through the different simulation layers (i.e., for each species, locus, or gene tree), with the possibility of specifying certain dependencies among parameters (i.e., with some parameters acting as hyper or hyper–hyper parameters) (Fig. 3).

Under the standard simulation workflow, *SimPhy* samples for each replicate genome-wide parameters, species-tree parameters, species-specific and gene family-specific rate variation parameters, and number of gene families. Genome-wide parameters control the expected distribution of duplications, losses, HGTs, and GC events across gene families. Species tree parameters include speciation and extinction rates, species tree height, number of taxa, relative distance of the out-group, number of individuals per species, effective population size, substitution rate, and generation time. For each gene family, specific duplication, loss, HGT, and GC rates are sampled from the distributions specified at the genome-wide level. The variation of the substitution rate across species, locus, and gene trees is also specified using statistical distributions at different levels, as detailed in the next section.

### Substitution Rate Variation

**Figure 4.**
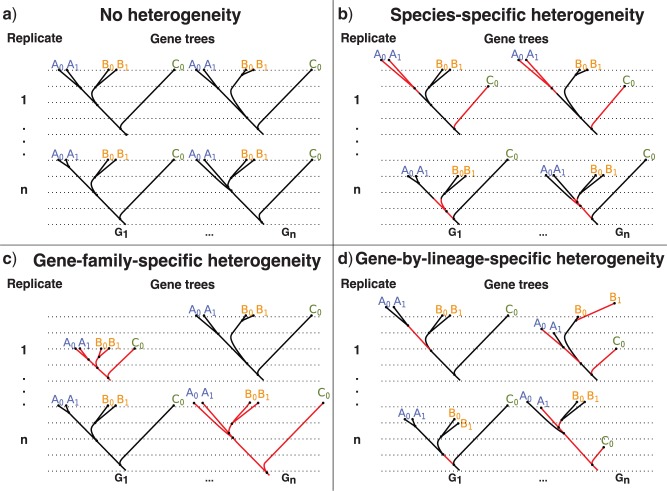
Sources of substitution rate heterogeneity among lineages available in *SimPhy*. Each subfigure represents the same simulation scenario but with different types of rate variation. Branches affected by rate variation are highlighted with a different color. Each simulation scenario consists of two independent replicates (rows) of two gene families (columns) in three species (A, B, C) with five individuals (${\text{A}}_{0}$, ${\text{A}}_{1}$, ${\text{B}}_{0}$, ${\text{B}}_{1}$, ${\text{C}}_{0}$). Subfigure a) shows trees simulated without substitution rate heterogeneity—hence ultrametric—with topologies and branch lengths generated by the coalescent process. Subfigure b) depicts a case of species-specific heterogeneity, where changes in rate affect whole ancestral/contemporary species genome-wide (e.g., for every gene family in replicate 1 species A branches evolve faster and species C branches slower). Subfigure c) is an example of gene family-specific heterogeneity, where rate changes affect the total history of specific gene families (note that in this case ultrametricity still holds) (e.g., in replicate 1, gene family (${\text{G}}_{1}$) evolves much slower than gene family (${\text{G}}_{n}$)). Subfigure d) shows gene-by-lineage-specific heterogeneity, where changes in rate affect particular gene tree branches independently (e.g., in replicate 1, gene family (${\text{G}}_{1}$), the ancestral A lineage evolves faster). In addition, the level of heterogeneity can also be modulated among replicates (e.g., in subfigure (c), replicate 1 shows much more heterogeneity than replicate n).

The generative model of *SimPhy*, being based on coalescent and birth–death models, is in principle intrinsically ultrametric and, therefore, follows a strict molecular clock. However, it is well known that many real data sets deviate from a strict molecular clock (see Ho et al. 2011). We have, therefore, implemented in *SimPhy* different sources of rate variation among lineages—lineage-specific, gene family-specific and gene-by-lineage-specific (Fig. 4)—which modify the branches of the gene trees. In all cases a rate multiplier is sampled from a Gamma distribution, whose mean is forced to be one to preserve the mean substitution rate, and that therefore can be parameterized by a single *alpha*-shape parameter. Since these multipliers are independently sampled, this approach is equivalent to the use of uncorrelated relaxed clocks (Drummond et al. 2006).

#### Species-specific substitution rate heterogeneity

A rate multiplier is sampled for each branch of the species tree, modeling how different species evolve at different speeds—for example, due to ecological conditions, metabolic rates (Martin and Palumbi 1993) or DNA repair efficiency (Britten 1986). The *alpha* parameter can be fixed or sampled *de novo* for each species tree (see section ‘Distribution-Driven Parameterization’).

#### Gene family-specific substitution rate heterogeneity

A rate multiplier is sampled for each locus tree (i.e., multiplying all the branches of a given locus tree), modeling how different gene families can evolve at a different pace—for example, due to functional constraints (Li 1997). The *alpha* parameter can be sampled independently for each species tree.

#### Gene-by-lineage-specific substitution rate heterogeneity

A rate multiplier is sampled for each branch of the gene tree, modeling how different gene tree branches evolve at different speeds due to gene–lineage interactions—for example, due to selective bursts (Wallis 1994). The *alpha* parameter can be fixed or hierarchically sampled across the different simulation layers (e.g., the most complex parameterization would sample a hyper–hyper parameter for each species tree, a hyper parameter for each locus tree and an alpha parameter per gene tree).

The baseline substitution rate is measured in number of substitutions per site per generation. Nevertheless, *SimPhy* also takes into account absolute time units and, therefore, incorporates a generation time parameter. To increase flexibility, species-specific generation times can also be specified (see Kohne 1970).

Note that if the final objective is the simulation of multiple sequence alignments, a fifth layer of rate variation, in this case among sites (Yang 1996), can be specified using INDELible.

### Input and Output

*SimPhy* has been implemented in C99 as a non-interactive command line program. Input parameters can be given in the command line or specified in a configuration file. Users can also provide their own species or locus trees in a Nexus file. The program will output more or less information depending on a verbosity level controlled by the user. Output files include a species tree file, a locus tree file with all the locus trees, and one gene tree file per locus tree (typically containing a single gene tree). Depending on the settings, the program can also print out the species tree/locus tree mappings and the locus tree/gene tree mappings. Moreover, *SimPhy* can also generate a SQLite3 database with all the simulation parameters per species, locus, and gene tree and some extra statistics. SimPhy's gene trees can be used to generate multiple sequence alignments in principle with any sequence simulator. To help in this task, the *SimPhy* package includes a script for directly running one of these simulators—INDELible (Fletcher and Yang 2009)—in a subsequent step.

## Validation

We performed a series of validation experiments, described below, to test that *SimPhy*'s output fits different theoretical or numerical expectations. The entire validation was conducted using in-house Bash, Perl, and R scripts (using APE [Paradis et al. 2004] and phytools [Revell 2012]) available as Supplementary Material on Dryad at http://dx.doi.org/10.5061/dryad.707td. In addition, the whole simulation process was also carefully checked by hand in the debugging developing stage on several simple scenarios.

### Species Tree Simulation

For the SSA algorithm, we assayed the Yule process with 10 different birth rates (λ) (from 2.67 to 6.67 speciations/1M generations) using 10,000 replicates per level and a fixed tree origin (t) (1M generations), and compared the observed mean number of leaves with its theoretical expectation $E(N(t))=2e^{\lambda t}$ (Mooers et al. 2012; Supplementary Fig. S1 available as Supplementary Material on Dryad at http://dx.doi.org/10.5061/dryad.707td). For the BDSA, we assayed 100 combinations of 10 different birth rates (from 2.67 to 6.67 speciations/1M generations) and 10 taxa sizes (from 50 to 500 species), using 10,000 replicates per combination, and compared the mean tree height with the one obtained using *TreeSim* (Stadler 2011; Supplementary Fig. S2).

### Locus Tree Simulation

We validated the observed average gene family size –i.e., after discarding losses and superfluous branches, $E(N(t))=\frac{le^{(\lambda -\mu )t}}{1-(p0m^{l-2})}$; $p0m=\frac{\mu (1-e^{-(\lambda -\mu )t})}{\lambda -\mu e^{-(\lambda -\mu )t}}$. We explored 125 parameter combinations of five duplication rates (λ) (from 6.67 to 33.3 duplications/10000M generations), five loss rates (μ) (from 0.5λ to 0.167λ) and five number of species (l) (from 50 to 250 species), with a fixed species tree height (t) (1000M generations) and 10000 replicates per combination (Supplementary Fig. S3). In addition, since gene family size is not affected by HGT or GC, we tested the expected number of HGT events E(T(l))=hl under 25 parameter combinations of five different HGT rates (h) (from one to 20 transfers/1M generations) and five locus tree lengths (l) (from 42 to 260 M generations), with 10000 replicates per combination (Supplementary Fig. S4).

### Gene Tree Simulation

We validated the simulation of the multispecies coalescent comparing the time to the most recent common ancestor (TMRCA) obtained with *SimPhy* and DendroPy (Sukumaran and Holder 2010) on 50 different scenarios (number of species sampled from a Uniform(50,500), speciation rate of one speciation/1M generations and effective population size sampled from a Lognormal(14,0.4)) with a total of 10,000 gene trees (Supplementary Fig. S5). The multilocus coalescent process was validated using a similar approach, comparing the average TMRCAs of the subtrees modeled by the bounded multispecies coalescent process obtained with *SimPhy* and DLCoal_sim (fixed species tree, five locus trees, duplication rate 0.2 duplications/1M generations and effective population size 1M individuals) with a total of 10,000 gene trees (Supplementary Fig. S6).

All the validation checks were clearly successful, showing only negligible deviations from the expectations or when compared to other simulations programs in spite of the large variance induced by the different evolutionary processes implemented.

## Benchmarking

We carried out four experiments to characterize *SimPhy*'s computational efficiency and scalability, where central processing unit (CPU) time was defined as the sum of user and system time returned by the Berkeley Software Distribution (BSD) time command. For scenarios without HGT or GC, we also computed the running times of DLCoal_sim (Rasmussen and Kellis 2012). For simplicity we used a classic grid-like parameterization with 10 replicates per scenario and one individual per species. All the output options of *SimPhy* were active. Errors and running times over 300 seconds were treated as missing data. All the analyses were run in a MacBook Pro Intel Core i7 2.3Ghz, 8GB of RAM, and a Solid State Drive. A generalized linear model with a Gamma error distribution was fitted to the resulting data to assess the relationship between running times and the variables studied. All the scripts used to carry out this benchmarking are available as Supplementary Material on Dryad at http://dx.doi.org/10.5061/dryad.707td.

### Benchmark 1

**Figure 5.**
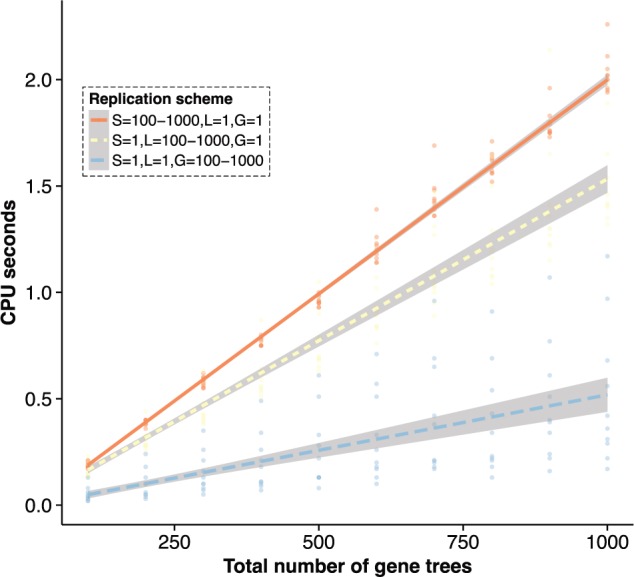
*SimPhy*'s running time against the number of gene trees simulated. A generalized linear model with a Gamma error distribution and the identity function as link was fitted to each data series. See section ‘Benchmark 1’ for the simulation details.

The first benchmark was designed to check the general performance and scalability of *SimPhy* with an increasing number of gene trees simulated under the joint effect of ILS, GDL, HGT, and GC (Fig. 5). We simulated 30 different scenarios under three different schemes (#species trees/#locus trees per species tree/#gene trees per locus tree): 1/1/100-1000, 1/100-1000/1 and 100-1000/1/1. For each scenario, we simulated 50-taxon species trees with a tree height of 1M generations, a speciation rate of 0.00001 speciations/generation, and an effective population size of 10000. We specified moderate but equal duplication, loss, HGT, and GC rates (0.5 events/1M generations). *SimPhy* is extremely fast and scales linearly with the number of trees (Fig. 5). For example, it generates 1000 gene trees for 50 species under a complex model with ILS, GDL, HGT, and GC in less than 2 s.

### Benchmark 2

This benchmark was intended to compare *SimPhy* and DLCoal_sim   under a very simple model, with only moderate levels of ILS. We designed 10 scenarios with 10 numbers of species, varying from 50 to 500. For each scenario, we simulated a single species tree, assuming a speciation rate of 1 speciation/1M generations, an effective population size of 10,000, and generated 100 gene families. *SimPhy* scales linearly with the number of species, while DLCoal_sim only does the same for small species trees, being unable to complete simulations with more than 300 species (Supplementary Fig. S8). Remarkably, *SimPhy* was at least one order of magnitude faster than DLCoal_sim. This might be explained by the fact that *SimPhy* differentiates subtrees that can be modeled by the multispecies coalescent from those that require the bounded alternative, using a much faster and less error-prone algorithm for the former situation. Moreover, in the absence of GDL, HGT, or GC, locus trees and species trees are equivalent, so in these cases *SimPhy* directly uses the species trees as locus trees instead of simulating them *de novo*. In addition, *SimPhy* and *DLCoal_sim* also have important implementation differences—for example, data structures, coding language—which make *SimPhy* faster in most scenarios. Finally, *SimPhy* uses a multiple precision library for very complex scenarios to favor its scalability.

### Benchmark 3

In this case, we characterized *SimPhy*'s and DLCoal_sim's running times for a model with moderate levels of ILS and GDL. We designed 30 scenarios, with 100, 200, and 300 species and 10 duplication rates from 1 to 3.25 duplications/1M generations. For each scenario, we simulated a single species tree, assuming a speciation rate of 0.1 speciations/1M generations an effective population size of 10,000, and generated 100 gene families. Logically, duplications increased the running time for both programs, but with *SimPhy* still being much faster than *DLCoal_sim* (Supplementary Fig. S9). Moreover, *DLCoal_sim* showed again important scalability problems, being unable to simulate trees with 300 species.

### Benchmark 4

**Figure 6.**
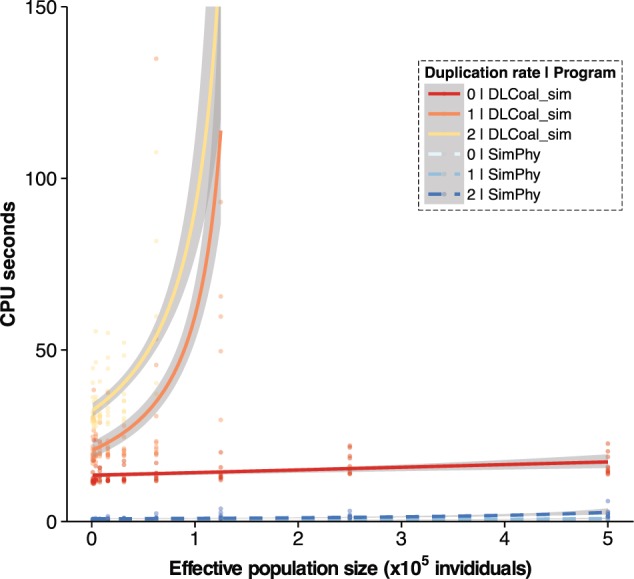
Running time comparison between *SimPhy* and DLCoal_sim under a GDL+ILS model. Scatter plot of the time needed by the two simulators to generate 100 gene trees from 100 locus trees using three different duplication rates (duplications/1M generations) under different effective population sizes. A generalized linear model with a Gamma error distribution and the inverse function as link was fitted to each data series. DLCoal_sim's execution times over 150 s are not shown.

Here we aimed to evaluate the sampling efficiency of the multilocus coalescent model by exploring a model with various levels of GLD and ILS. We designed 30 scenarios with 10 effective population sizes—from 976 to 500,000 individuals—and three duplication rates—0, 1, and 2 duplications/1M generations. For each scenario we simulated a single 100-taxon species tree, assuming a speciation rate of 10 speciations/1M generations and a tree height of 0.5 M generations, and generated 100 gene families. For both programs we observed an important increase in execution time with higher ILS in the presence of duplications, and almost no effect in their absence (Fig. 6). Importantly, *SimPhy* was again at least one order of magnitude faster and scales much better than *DLCoal_sim*. Several *DLCoal_sim* replicates were allowed to run beyond the 300 s limit but did not finish even after 24 h. This behavior is likely reflecting a problem in the rejection sampling algorithm that *DLCoal_sim* uses to sample the bounded multispecies coalescent (specifically the lineage counts), since this algorithm is prone to get stuck sampling highly improbable scenarios. On the other hand, *SimPhy* uses an alternative sampling strategy (see section ‘Simulation Process’ and Online Appendix 1) that seems to effectively mitigate this problem.

## Test-Case Study: Duplication Time Overestimation of DL Reconciliations

**Figure 7.**
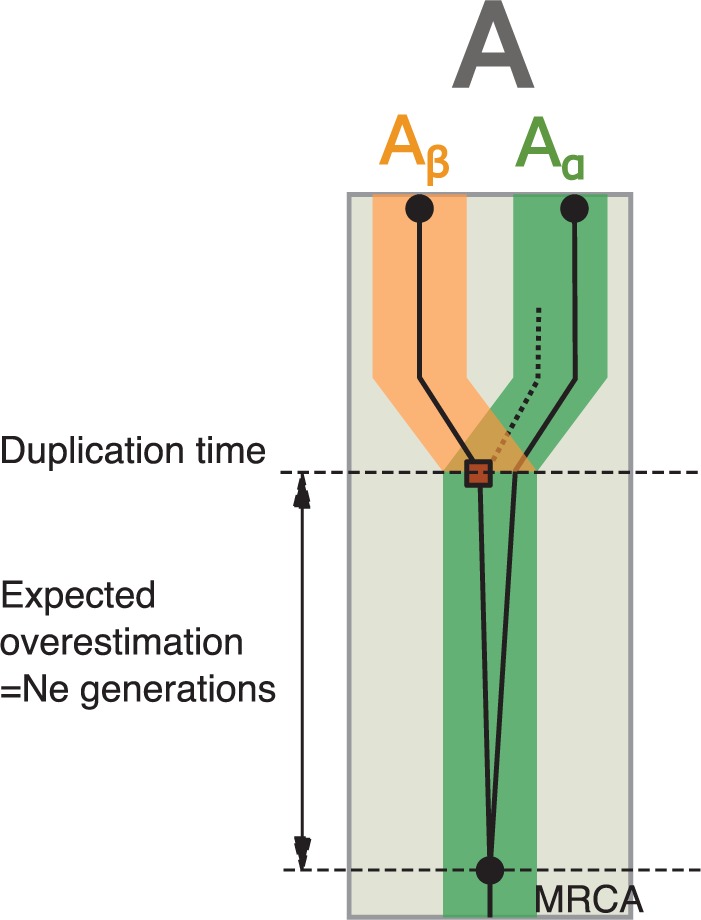
Expected overestimation of the time of gene duplication induced by species tree/gene tree reconciliation methods that do not consider locus trees. The figure represents the evolutionary history and expected overestimation of the duplication time for two paralogous copies (${\text{A}}_{\alpha }$, ${\text{A}}_{\beta }$) in a single individual (A). The gene tree (thin black lines) evolves inside the locus tree (medium-thick lines) along a species tree branch (light gray shadow in the background). The duplication takes place in the gene copy represented by a square, whose lineage in the original locus does not reach the present (dashed line). For haploid populations, the expected overestimation is *Ne* generations.

We have previously shown that the most recent common ancestor (MRCA) of a new gene originated by duplication and its paralog does not necessarily coincide with the individual where this duplication first occurred, generating a systematic overestimation of the duplication time for locus tree unaware reconciliation methods (Mallo et al. 2014b; Fig. 7). Here we further explored this issue by quantifying this overestimation in more complex scenarios for which theoretical expectations are not available. To do so, we simulated 1,000,000 replicates with a number of species uniformly distributed from 25 to 200, with a fixed speciation rate (10 speciations / 1M time units), an effective population size uniformly distributed from 1000 to 10,000 individuals, and with just one gene family per genome. The locus tree simulation was parameterized with a duplication rate sampled from a Uniform distribution of 0.05 to 0.5 events/1M generation, and with a number of individuals per species uniformly ranging from one to five. To check the relationship between the simulated parameters and the overestimation bias, we performed a stepwise selection under the Akaike information criterion (AIC) of a generalized linear model with Gamma error distribution and inverse link on a linear combination of all the simulation parameters. As dependent variable we used the mean of the distance between the real duplication node (locus tree) and the first coalescence between lineages coming from the two different paralogs for all the duplications for each simulation replicate.

Our results show that the effective population size increases the overestimation bias, while the number of individuals per species and the duplication rate have the opposite effect, being the only three parameters retained in the best-fit linear model. That a larger effective population size increases the overestimation bias is expected, as the coalescent time of any two lineages grows with it. An increasing number of individuals per species should reduce this bias, as with more lineages going through the duplication node the time for the first coalescence among paralogs decreases. The effect of the duplication rate, inversely correlated with this bias, is less clear. Nevertheless, it can be tentatively explained by the coalescent bounds imposed by duplications. Expected coalescent times along a bounded subtree are shorter than along an unbounded subtree, since the probability of coalescence is scaled by the probability of the TMRCA being less or equal to the bound (i.e., we can think of the unbounded coalescent as a bounded coalescent with the bound in the infinite, and therefore scaled by one). Thus, the bigger the duplication rate, the bigger the probability of having duplications in bounded subtrees, and the bigger the reduction on the expected duplication time overestimation generated by the coalescent bounds.

Interestingly, we can conclude that the expected overestimation for the simplest case—2Ne for one diploid individual with two paralogs; (Mallo et al. 2014b)—constitutes an upper bound, with a smaller bias expected in more complex scenarios (with duplications and multiple individuals per species). Finally, we also note that the size of the species tree did not seem to play a significant role in this case.

## Conclusion

We have introduced, validated, and benchmarked *SimPhy*, to our knowledge, the first software that simulates gene tree family evolution under the three main evolutionary processes that generates species tree/gene tree incongruence—ILS, GDL, and HGT. This, together with its comprehensive heterogeneity models and the parameter sampling strategy should help *SimPhy* becoming a powerful phylogenomic tool. We have also conducted a simple case study to show a potential application (duplication time overestimation), but we envision many more to come. In fact, *SimPhy* has been already used to validate at least three species tree reconstruction methods (De Oliveira Martins et al. 2014; Bayzid et al. 2015; Mirarab and Warnow 2015).

## Availability

*SimPhy* is distributed under the license GNU GPL v3. It is written in C and it relies on four libraries, the GNU Scientific Library (GSL), the GNU Multiple Precision Arithmetic Library (GMP), the GNU MPFR Library, and SQLite3. Users can find the source code, precompiled executables, a detailed manual and example cases on a GitHub repository (https://github.com/adamallo/SimPhy, last accessed November 17, 2015). We provide two flavors of precompiled executables, for Linux—which passed 73 out of 74 tests for the 64 bits version and 84 out of 87 tests for the 32 bits version under the Linux Standard Base distribution checker—and MacOSX systems.

## Supplementary Material

Data available from the Dryad Digital Repository: http://dx.doi.org/10.5061/dryad.707td.
